# Regionally and climatically restricted patterns of distribution of genetic diversity in a migratory bat species, *Miniopterus schreibersii *(Chiroptera: Vespertilionidae)

**DOI:** 10.1186/1471-2148-8-209

**Published:** 2008-07-18

**Authors:** Raşit Bilgin, Ahmet Karataş, Emrah Çoraman, Todd Disotell, Juan Carlos Morales

**Affiliations:** 1 Institute of Environmental Sciences, Boğaziçi University, Bebek 34342, Istanbul, Turkey; 2 Department of Ecology Evolution and Environmental Biology, Columbia University, New York, NY, 10027, USA; 3 Center for Environmental Research and Conservation, Columbia University, 1200 Amsterdam Avenue, MC 5557, New York, NY, 10027, USA; 4 Niğde Üniversitesi, Zübeyde Hanım Sağlık Yüksekokulu, 51100 Niğde, Turkey; 5 Department of Anthropology, New York University, 25 Waverly Place, New York, NY 10003, USA; 6 California State University, Stanislaus, One University Circle Turlock, CA 95382, USA

## Abstract

**Background:**

Various mechanisms such as geographic barriers and glacial episodes have been proposed as determinants of intra-specific and inter-specific differentiation of populations, and the distribution of their genetic diversity. More recently, habitat and climate differences, and corresponding adaptations have been shown to be forces influencing the phylogeographic evolution of some vertebrates. In this study, we examined the contribution of these various factors on the genetic differentiation of the bent-winged bat, *Miniopterus schreibersii*, in southeastern Europe and Anatolia.

**Results and conclusion:**

Our results showed differentiation in mitochondrial DNA coupled with weaker nuclear differentiation. We found evidence for restriction of lineages to geographical areas for hundreds of generations. The results showed that the most likely ancestral haplotype was restricted to the same geographic area (the Balkans) for at least 6,000 years. We were able to delineate the migration routes during the population expansion process, which followed the coasts and the inland for different nested mitochondrial clades. Hence, we were able to describe a scenario showing how multiple biotic and abiotic events including glacial periods, climate and historical dispersal patterns complemented each other in causing regional and local differentiation within a species.

## Background

**Figure 1:**
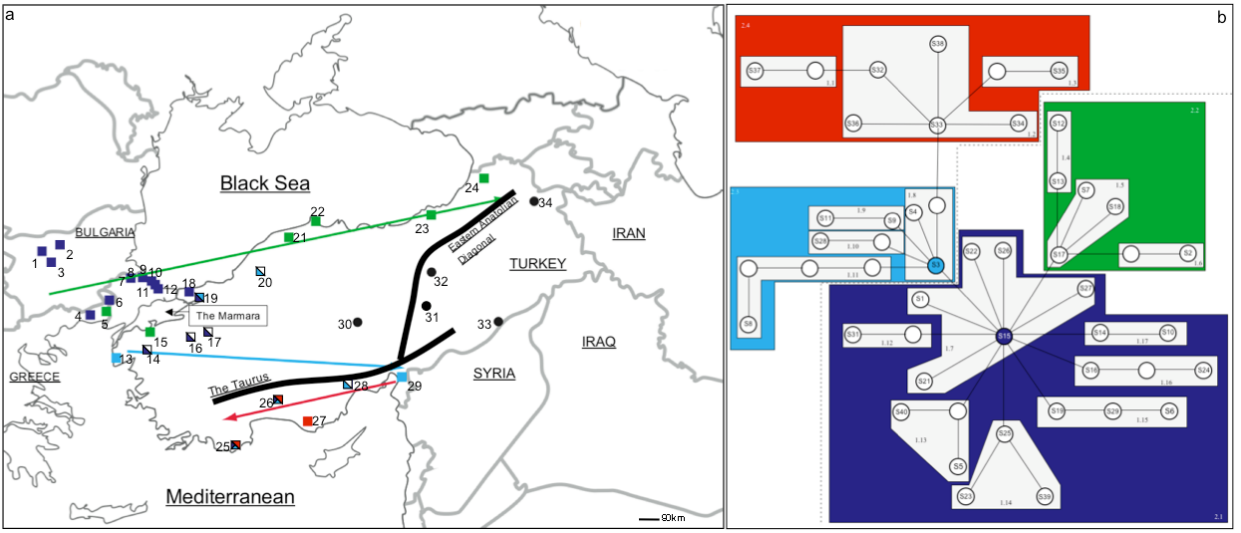
**a) The geographic positions of the area of interest. The squares and circles depict the localities that have individuals with clade S and clade P haplotypes, respectively**. The details of the sampling localities are provided in Table 1. The colors of the squares correspond to 2^nd ^level nested clades in Figure 1b. Specifically, the purple squares designate the distribution of the putative most ancestral haplotype, S15, of the entire network. The light blue squares designate the most ancestral haplotype, S3, in the light blue 2^nd ^level nested clade, 2.3. The green squares represent all of the haplotypes comprising green 2^nd ^level nested clade, 2.2. When the squares comprise more than one color, they represent haplotypes comprising purple or light blue 2^nd ^level nested clades, other than S15 or S3, being found in the particular locality. For instance, the purple/white coloring of localities 14, 15 and 16 indicate that haplotypes belonging to the purple nested clade, 2.1, are found in it, but none of these are the haplotype S15. Similarly, in localities 20 and 28, designated as light blue and white, haplotypes belonging to the light blue nested clade, 2.2, are found, but none of these are the haplotype S3. In locality 19, haplotypes belonging to the purple (but not S15) and light blue (but not S3) are found. Finally in localities 25 and 26, in addition to the red 2^nd ^level nested clade, 2.4, haplotypes, non-S15 purple and non-S3 light-blue haplotypes are found. b) NCA diagram for clade S. The colored polygrams represent the four, second-level nested clades.

The geographic transitions between continents can result in species diversification and endemism, forming regions of allopatry, and contact zones for divergent flora and fauna. Sulawesi, for instance, an island located between Continental Asia and Australia, has elements of both faunal assemblages within its boundaries, hosting an elevated number of species and endemics [1]. Mexico, as another example, being located in a transition zone between tropical central America, and temperate North America is considered to be a megadiversity country [2]. Within the temperate zones, southeastern Europe and Anatolia are located within a similar geographical transition centered in between Europe, Asia and Africa (Figure 1a). As such, this region comprises an interesting area for investigation as a zone of allopatry with geographical barriers to gene flow [3,4], a contact zone for divergent biota from different continents and climatic regimes, and a refugium for the entire western Palearctics [5,6].

In this study, we examined the nuclear and mitochondrial genetic structure of the bent-winged bat, *Miniopterus schreibersii*, in southeastern Europe and Anatolia. *M. schreibersii *is a colonial, cave-dwelling [7], and polytypic species with one of the largest Old World ranges among mammals. Its global distribution spans the Palearctics, Africa and Australia, with 17 subspecies described [8,9]. Recent studies showed high levels of nuclear and mitochondrial genetic structure in a congeneric species, *Minipterus natalensis *in South Africa [10]. Differentiation has also been reflected in taxonomy of this species in Australia [11], and southeastern Europe and Anatolia [12], where three and two subspecies have been described, respectively.

Observing the genetic differentiation in this species in southeastern Europe and Anatolia, our goal was to try teasing apart the differential contribution of various factors that can contribute to its genetic differentiation. Topographical features in the region, including the Taurus Mountains, the eastern Anatolian Diagonal Mountain Chain and the Sea of Marmara (Figure 1a) are potential barriers to gene flow that could result in allopatric differentiation of populations. For instance, The Marmara was proposed as a potential barrier preventing the secondary contact of isolated refugial populations in another bat species, the greater horseshoe bat [13]. The region is also interesting due to different climates regimes that co-exist, as differential adaptations to these could promote population divergence. In addition to drastic differences in precipitation between the coastal and inland regions, there also exist regional differences in climate. For instance, the Mediterranean coast, the Black Sea coast and the Marmara region all show slight differences in terms of temperature and precipitation patterns [14]. The glacial history of the region, especially at the Pleistocene, is another candidate for having caused divergence of populations. The Balkans is one of the main glacial refugial areas for the entire Europe [15,16], and the Caucasus is an important refugium for western Asia and the Middle East in general [17,18]. Anatolia is located in between these two major refugial areas, and this proximity could have left a signature on the genetic make-up of populations. To address these questions, we used nuclear microsatellite markers, as well as mtDNA markers, comprising the next logical step to the previous study of Bilgin et al. [12], which had used only mtDNA data to mainly evaluate the taxonomic status of *M. schreibersii *in this region. In the end, we were able to construct a scenario outlining the historical details of the genetic differentiation of *M. schreibersii *in the transition between Europe and Anatolia.

## Methods

### Sampling

121 individuals of *M. schreibersii *were sampled in 34 locations in Bulgaria, Greece and Turkey (Figure 1a and Table 1), spanning a range of about 1700 km. This comprises an increase from 58 individuals in 14 locations that was used in the previous study of Bilgin et al. [12], on the same species. The caves where samples from Bilgin et al. [12] were used in the current study are indicated in Table 1. Fieldwork was undertaken, in the years 2004 and 2005, in August, to avoid disturbing the nursery colonies. Through inspection of the degree of fusion of the phalangeal epiphyses, the adult status of the sampled bats was confirmed, and no juvenile bats were sampled [19]. Tissue for genetics analysis was collected from each of the wing membranes (plagiopatagium) of individual bats by using biopsy-punches (3 mm diameter) as outlined by Worthington Wilmer and Barratt [20]. The 3 mm holes in each wing are known to heal in approximately four weeks.

**Table 1:** The names of the sampling locations, their ID's as they appear in Fig 1., the geographical coordinates, sample size per locality (*N*), number of haplotypes, the names of the haplotypes as they appear in Figures 1 and 2, and the numbers of males and females at each site.

Locality ID	Locality Name	Latitude	Longitude	*N*	No. of Haplotypes	Haplotype names (numbers)	No. of Males (M) and Females (F)
1	**Parnitzite**	43.2003	24.4321	10	7	S6, S15(3), S24, S25, S29(2), S31, S39	8 M, 2 F
2	**Devetashkata**	43.2337	24.8852	5	4	S1, S5. S23. S25 (2)	2 M, 3 F
3	**Mandrata**	43.0620	24.52469	2	2	S25, S29	2 M
4	**Maronia**	40.9321	25.5041	2	2	S15, S19	2 F
5	**Koufovouno**	41.3514	26.4862	6	3	S15(4), S25, S26	4 M, 2 F
6	**Tsout**	41.3514	26.4862	3	2	S13(2), S15	1 M, 2 F
7	**Kuru**	41.8397	27.5574	1	1	S15	1 M
8	**Kiz**	41.8363	27.5573	4	4	S15, S16, S39, S40	4 M
9	**Kiyikoy**	41.6132	28.1124	3	1	S15(3)	1 M, 2 F
10	**Cilingoz**	41.5238	28.2216	6	3	S15(4), S25, S26	4 M, 2 F
11	Kovantasi	41.7072	27.9112	4	3	S14, S15(2), S26	3 M, 1 F
12	Kocakuyu	41.2885	28.318	1	1	S15	1 M
13	Canakkale	40.0241	26.1123	1	1	S3	1 M
14	Havran	39.348	27.09	1	1	S22	1 M
15	Balikesir	40.2314	27.5244	1	1	S12	1 M
16	Bursa	40.1058	29.0042	1	1	S14	1 F
17	Bursa2	41.1906	31.4722	1	1	S14	1 M
18	Sofular	41.1853	29.5121	1	1	S15	1 M
19	Gokceoren	40.8496	29.9956	2	2	S9, S27	2 M
20	Sakarya	40.5743	30.2042	1	1	S9	1 F
21	Kastamonu	41.5795	33.7673	1	1	S17	1 M
22	Sinop	41.9383	35.09467	1	1	S7	1 M
23	Catak	40.76479	39.563	1	1	S17	1 M
24	Cehennemdere	41.3703	42.0126	8	3	S2(2), S17(5), S18	4 M, 4 F
25	**Hidirellez**	36.17276	29.64448	14			10 M, 4 F
26	**Catdere**	37.15961	31.812	14	6	S8, S11(2), S21, S32, S33(8), S36	10 M, 4 F
27	Yalandunya	36.2198	32.4022	5	2	S33(2), S35(3)	2 M, 3 F
28	Anamur	36.0509	32.4928	1	1	S21	1 M
29	Hatay	36.5121	36.3113	1	1	S3	1 M
30	Armutdere	38.74014	34.9379	8	4	P2(3), P4(3), P5(1), P6(1)	2 M, 6 F
31	**Kayseri**	38.5824	35.2814	1	1	P3	1 F
32	**Kemah**	39.3531	39.0021	8	6	P2(2), P6(2), P7, P8, P9, P10	1 M, 7 F
33	Mardin	37.1844	40.4534	1	1	P1	1 F
34	Iğdır	40.0258	43.4004	1	1	P3	1 F

The localities where samples from Bilgin et al. [12] were used in the current study are shown in bold. From Kemah, only one individual was sampled in Bilgin et al. [12].

### Laboratory Methods

Mitochondrial DNAFor mtDNA analysis, the hyper variable region I of the control region (HV1), tRNA-proline and tRNA-threonine genes, and a partial cytochrome *b *sequence were sequenced. Half of a biopsy punch was used for each individual's DNA extraction, with a DNeasy Extraction Kit, following the manufacturer's protocols (QIAGEN, Valencia, CA). The primers C and E, and the PCR conditions were used as described in Wilkinson and Chapman [21] for DNA amplification. This was followed by cycle sequencing, both in 5' and 3' directions, using the primers C and E, respectively. This involved 25 cycles in 10 μl reactions, which were composed of 1 μl of PCR product, 5.7 μl of H_2_O, 0.3 μl of primer (20 μM), 1 μl of florescent dye (ABI Big Dye) and 2 μl of 5× buffer (provided with the florescent dye). The cycle sequencing parameters for each cycle were 10 seconds of denaturation at 96°C, 5 seconds of annealing at 50°C and 4 minutes of extension at 68°C, followed by a final extension of 7 minutes at 72°C. The sequencing reactions were cleaned using ethanol precipitation and run in an ABI 3730 automated sequencer following the manufacturer's protocol (Applied Biosystems, Inc.). The resulting sequences were assembled using Sequencher 4.1 (Gene Codes Corp.), and aligned using Clustal X [22] prior to further data analysis.

### Microsatellites

Four microsatellite primer pairs (Mschreib2, Mschreib3, Mschreib4 and Mschreib5) designed for *M. schreibersii *[10] and two pairs (D5S1457 and D6S271) designed for humans, which also amplify in baboons [23], were used to amplify and genotype six nuclear loci. The PCR amplifications were carried out with a QIAGEN Multiplex kit following the manufacturer's protocol. The reaction volume was cut down by a factor of five (10 μl instead of the manufacturer's recommended 50 μl) to decrease the overall cost, while 1 μl of template DNA was used. The optimal annealing temperature for each locus was found through a gradient PCR. The PCR reactions were successful at the annealing temperature of 57°C for all loci, except Mschreib2 and Mschreib4, which yielded amplicons at 61°C. A homozygote individual was sequenced for each locus using the respective forward and reverse primers, so as to confirm the identity of the repeat motifs with the published ones. The PCR products were run in an ABI Automated Sequencer 3730, using a Rox label (ABI, CA) to size the fragments, in a volume composed of 8.9 μl of High Dye (Applied Biosystems, Inc.), 0.1 μl of Rox, and 1 μl of PCR product for each sample. The final sizes of the fragments were computed in GeneMapper (Applied Biosystems, Inc.) and scored manually. The homozygous individuals were amplified and scored three times to avoid allelic dropout and confirm allele sizes; as for the heterozygous individuals, they were amplified and scored until the sizes matched in two separate runs.

### Data analysis

#### Mitochondrial DNA

Basic descriptive statistics and genetic diversity parameters such as haplotype diversity (h), nucleotide diversity (π) and number of polymorphic sites [24,25], were calculated in DnaSP 4.20.2 [26]. These calculations were also made for the reciprocally monophyletic clades S and P, see below, separately. We reconstructed the phylogeny of the haplotypes using distance (neighbor-joining, NJ) and maximum parsimony analyses, and the software PAUP* 4.06b [27]. Nodal support for NJ and maximum parsimony trees were evaluated by 1000 bootstrap replicates. The substitution model to be used for building the distance matrix was selected by applying a likelihood ratio test for the goodness of fit of various substitution models to the data by using the program Modeltest 3.7 [28]. For the neighbor joining tree, the model of nucleotide evolution, selected by Modeltest, which fit the data best was that of Hasegawa et al. [29] [(HKY85) + Γ [30], shape parameter = 0.07] and was used in the construction of the NJ tree. In the maximum parsimony analysis, TBR (tree bisection and reconnection) was used as the branch-swapping algorithm as it is the most effective routine for recovering an optimum set of cladograms [31]. Gaps were treated as missing data. The sequences were checked for any evidence for saturation, using percent genetic divergence versus transition/transversion plots in DAMBE [32]. When there was evidence for saturation, the parsimony analysis was adjusted using a step matrix by weighting transversions inversely against transitions, based on their frequency. The ratio of transitions to transversions was calculated following Nei and Kumar [33] in MEGA4 (Tamura et al., 2007). MEGA4 was also used to compute the genetic distances between clades S and P, using maximum likelihood composite distances. Average number of substitutions *per *site (D_xy_) was calculated using DnaSP 4.20.2.

Differentiation in mtDNA was also explored using analysis of molecular variance (AMOVA) [34], by calculating the F_st _analogue Φ_st_. The program ARLEQUIN 2.0 [33] was used to make the AMOVA calculations for mtDNA. The AMOVA calculations included two separate groupings. One of these was based on grouping clades S and P as separate populations. The second set of regional groups was prepared to test for the effect of geographical barriers to gene flow. These included four groups, which comprised sampling sites to the west of the Marmara Sea (sites 1–18 in Figure 1), between the Marmara Sea, the Taurus and the eastern Anatolian Diagonal (sites 13–24, 30), to the south of the Taurus (25–29), and to the east of the eastern Anatolian Diagonal (sites 31–34).

Nested clade analysis (NCA) was used to determine the causes of intraspecific structure, if any. The program TCS [35] was used to build a statistical parsimony network and subsequently GeoDIS 2.0 [36] was used for making an NCA. We used the inference key of 11/11/2005 from http://darwin.uvigo.es/download/geodisKey_11Nov05.pdf

Evidence for population expansion was explored using the neutrality test statistics *R*_2 _[37] and *F*_*S *_[38] in DnaSP. Based on simulation models, among various statistics that determine signatures of past population growth, *R*_2 _and *F*_*S *_are the two that have the greatest statistical power [35]. The significance of these statistics was assessed with 1000 coalescent simulations. In addition, Bayesian Skyline plots [39] were used to detect changes in effective population sizes in the past. We estimated the divergence times of reciprocally monophyletic clades using a Bayesian coalescent approach implemented in BEAST 1.4.6. [39]. The HKY + Γ, as revealed by Modeltest, were used as the substitution and site heterogeneity models, respectively. Again, based on Modeltest, The Γ prior was set to 0.07. The tree model prior was determined by using a UPGMA tree to construct an initial tree, and the mean rate and covariance priors were set to uniform values. The chain was run for 100,000,000 generations, making sure that the ESS values of each statistic was at least 1000. Convergence was examined in TRACER 1.4 http://beast.bio.ed.ac.uk/Tracer.

#### Microsatellites

Basic tests of Hardy-Weinberg equilibrium and linkage disequilibrium were carried out in FSTAT [40]. Sequential Bonferroni corrections were made to correct for levels of significance for multiple tests [41]. The software Micro-Checker [42] was used to test for the presence of stutter bands, large allelic drop-out, and null-alleles. The unbiased number of alleles (based on sample size) was calculated in FSTAT, using a method of rarefaction [43]. The expected and observed heterozygosities and their *P *values (for the entire data set, and separately for clades S and P), the uncorrected number of alleles, allelic diversity (in terms of effective number of alleles, N_e_), and frequency of alleles in clades S and P for each locus, are provided in the additional file. All of these computations were made using Genalex 6.1 [44]. In addition, corrected number of private alleles were calculated using a rarefaction method, and the software HP-rare [45]. Global and locus-by-locus AMOVA were made, and unbiased F_st _and R_st _estimators [of θ_st _[46] and ρ_st _[47], respectively] were also calculated using Genalex 6.1. Both F_st _and R_st _estimators were calculated to account for the possible underestimation of F_st _in detecting genetic differentiation when analyzing microsatellites [48].

## Results

### Mitochondrial DNA

For the analysis of mtDNA, 433 base pairs of mtDNA spanning cytochrome *b *(58 bp), tRNA-threonine (71 bp), tRNA-proline (66 bp) and HV1 (238 bp) were sequenced for 121 individuals. These sequences have been deposited to GenBank with the accession numbers EU332355–EU332392 and EU332393–EU332402. In this fragment, there were 52 polymorphic sites, 43 of which were parsimony informative. A total of 49 haplotypes were found. The haplotype diversity (h) was 0.926 (S.D. = 0.015) and nucleotide diversity (π) was 0.02593 (S.D. = 0.00327).

**Figure 2:**
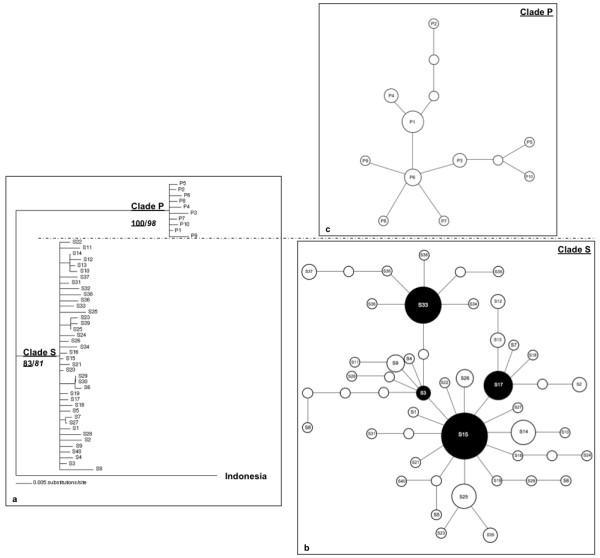
**a) Neighbor joining phylogenetic tree of mtDNA sequences for *M. schreibersii***. Bootstrap support values for clades S and P are underlined for the neighbor joining phylogram and in italics for the maximum parsimony tree. A sample of *M. schreibersii *from Indonesia was used as outgroup. **b) Statistical parsimony network of haplotypes (S1–S40) for clade S.** The unlabeled circles indicate hypothetical haplotypes. The size of each circle is proportional to the frequency of the particular haplotype in the sample.** c) The statistical parsimony network of haplotypes (P1–P10) for clade P.** The unlabeled circles indicate hypothetical haplotypes. The size of each circle is proportional to the frequency of the particular haplotype in the sample.

Phylogenetic trees for *M. schreibersii *were constructed using neighbor joining and parsimony methods (Figure 2a). A sample of *M. schreibersii *from Indonesia was included in the analysis as an outgroup. In the neighbor joining tree, two monophyletic clades have been identified, clade S and clade P, with high bootstrap support (83% and 100%, respectively). A heuristic maximum parsimony search also gave high bootstrap support to the nodes delimiting clades S (98%) and P (81%). In this analysis, the transversions were weighted against transitions based on their frequency (1:7). The C.I and R.I were both 0.908 for this tree.

In terms of descriptive statistics, a total of 38 haplotypes were found as belonging to clade S, in 102 individuals. Within clade S, there were a total of 37 polymorphic sites, 24 of which were parsimony-informative. Haplotype diversity was 0.932 (S.D. = 0.014) and nucleotide diversity was 0.00689 (S.D. = 0.00043). Within clade P, there were ten haplotypes, found in 19 bats, comprised of ten polymorphic sites of which six were parsimony-informative. Haplotype diversity was 0.897 (S.D. = 0.056) and nucleotide diversity was 0.0056 (S.D. = 0.00089).

The number of fixed and percent nucleotide differences between the haplotype groups is provided in Table 2, to give an idea of the extent of differentiation of groups. The percent divergence (modified from D_xy_) between clades S and P was 7.94%. The maximum composite likelihood distance between the clades was almost identical (0.078, S.E. = 0.015). The differences between the Indonesian sample and clades S and P were 12.19% and 8.29%, respectively. These differences between regions are indicative of differentiation when compared to the percent nucleotide differences within each haplogroup, which ranged between 0 and 0.68%.

**Table 2:** Percent differentiation and the number of fixed differences (in parentheses) between clades S, P, and the Indonesian sample.

	P	S	I
**P**	0.54 (0)		
**S**	7.94 (15)	0.68 (0)	
**I**	12.19 (38)	8.29 (23)	0% (0)

The diagonal comparisons indicate within-group differentiation.

The Bayesian estimate of the time of the split of the S and P clades was calculated as 233,000 years BP (95% Highest Posterior Density = 169,000–299,000 years BP). In these calculations, a molecular clock rate based on 20% *per *million years differentiation of D-loop in the noctule bat [49], which falls between the estimate for house mouse [10% *per *million years [50]) and bison (30% *per *million years [51]] was used.

Another mathematical treatment of the differentiation between clades S and P was made with an AMOVA. The pairwise Φ_st _was 0.92 and significantly greater than zero (*P *< 0.01, 1000 permutations). The percent of variance attributable to differences between clades was 92%, whereas this variance value within clades was 8% (Table 3). In terms of geographical distribution, both haplotypes belonging to clade S and P appeared on either side of any putative geographic barrier. In addition, using the tree approach, within either clade P or S, there didn't seem to be any geographically meaningful clusters. The AMOVA results, when made using the potential geographic barriers to define populations, showed that the percent of variation attributable to among group differences was 27% (Table 4).

**Table 3:** Percent distribution of variation when individuals are grouped as in clades S and P.

Hierarchical level	% variation	**d.f**.	*P *value
Amoung Groups	91.45	1	0.0
Amoung Populations within Groups	2.76	31	0.0
Within Populations	5.79	88	0.33

**Table 4:** Percent distribution of variation when individuals are grouped by geographical barriers.

Hierarchical level	% variation	**d.f**.	*P *value
Amoung Groups	27.10	3	0.0
Amoung Populations within Groups	56.58	29	0.0
Within Populations	16.31	88	0.04

Several additional analyses were made to detect the causes of this genetic structure within clades S and P by building a statistical parsimony network for each (Figure 2b and Figure 2c, respectively). Both of these networks showed star-like phylogenies, which is characteristic of expanding populations [35]. For clade S, haplotypes comprising nodes of expansion are colored in black (Figure 2b). A similar pattern of a star-like network was found for clade P, however there was only one potentially ancestral haplotype (P1, Figure 2c).

**Figure 3:**
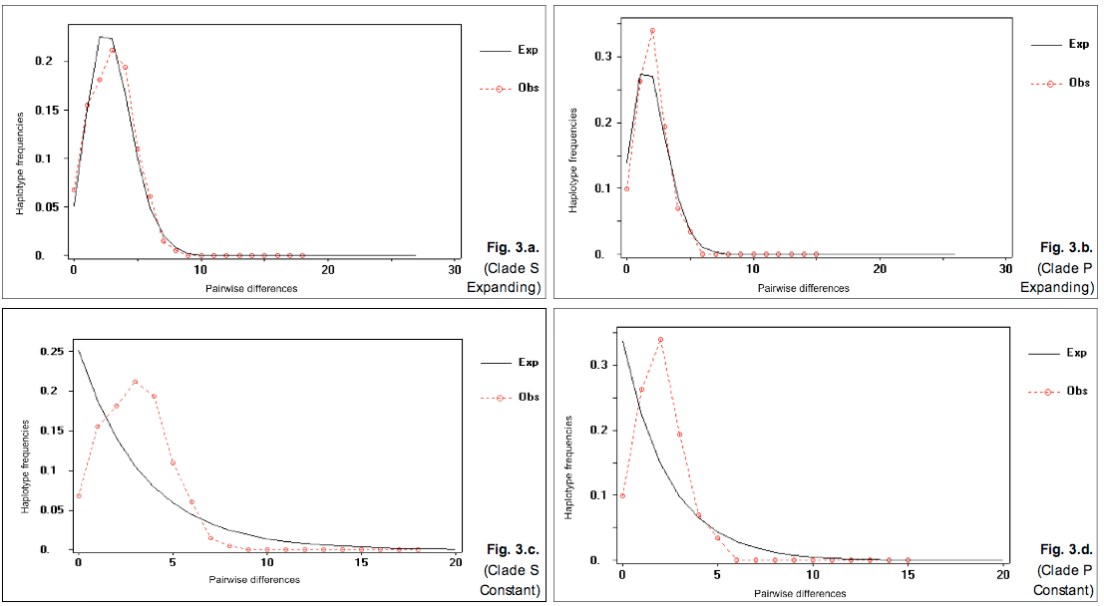
**a)** The observed and expected mismatch distributions in an expanding population model for clade S (*P *= 0.134). **b)** The observed and expected mismatch distributions in an expanding population model for clade P (*P *= 0.369). **c)** The observed and expected mismatch distributions in a constant size population model for clade S (*P *= 0.116). **d)** The observed and expected mismatch distributions in a constant size population model for clade P (*P *= 0.134).

**Figure 4:**
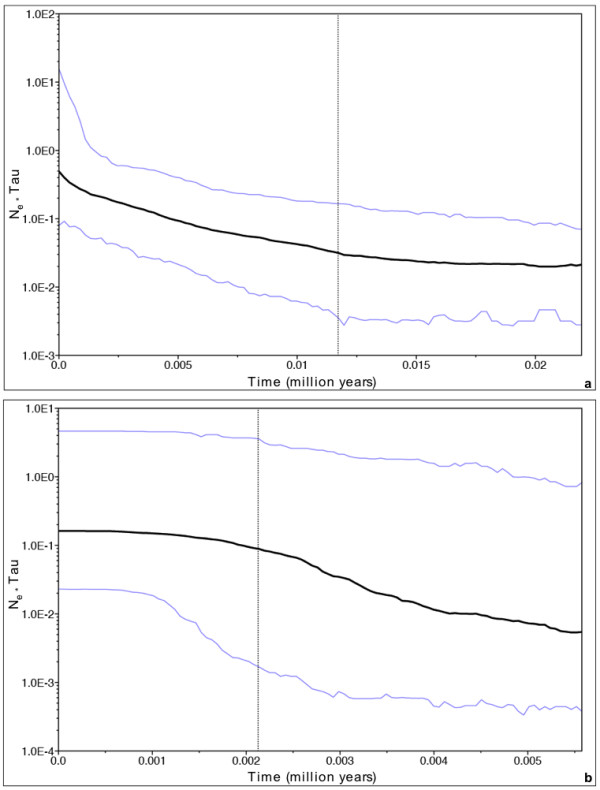
**a)** Bayesian skyline plot for clade S, including the 95% highest probability density interval.** b) **Bayesian skyline plot for clade P, including the 95% highest probability density interval.

Given this pattern of star-like networks, indicative of population expansion, the significance of these patterns was checked through the statistics *R*_2 _and *F*_*S*_. As the AMOVA indicated a significant genetic break between clade S and clade P, the analyses were run separately for each clade. For clade S, both of these statistics, whose values were 0.0385 and -32.2963, respectively, indicated significant expansions, as the probability of getting values lower than these was < 0.01 (1000 replicates). For clade P, the pattern was a similar one of significant *R*_2 _and *F*_*S*_, whose values were 0.0880 (*P *< 0.05) and -4.8960 (*P *< 0.01), respectively. Also the frequency of pairwise nucleotide differences of haplotypes was plotted with expectations under a constant-size model and a model of range expansion. The plots for both clade S and P fit a range expansion model better than a constant size model (Figures 3a–d). The Bayesian skyline plots for clades S and P also showed patterns of population expansion, initiating around 15,000 (Figure 4a) and 5,500 (Figure 4b) years ago, respectively.

Nested Clade Analysis (NCA) was applied to the statistical parsimony networks to infer if there were any geographic patterns within clades S and P. During the building of the nested cladogram, when a particular haplotype could not immediately be assigned to a nesting clade due to its position, it was included in the clade that had the smallest sample size, to increase the statistical strength of that nesting clade. In clade P, no significant associations between haplotypes were found and the hypothesis of panmixia could not be rejected. The nested cladograms for clade S showed four different second level nested clades (Figure 1b), with some significant associations. Based on expectations from the coalescent theory, the haplotype S15, within the purple nested clade (2.1), being the haplotype with the highest frequency, and the one that gave rise to the greatest number of haplotypes, was the most likely ancestral haplotype. This haplotype showed a distribution almost exclusively restricted to the Balkans (Figure 1a). The central haplotype (S3) of the light blue nested clade (2.3) budded off from the ancestral nested clade 2.1, and had a distribution along the Mediterranean coast. The red nested clade (2.4), which was directly connected to S3, was exclusively found along the Mediterranean coast. The green nested clade (2.2), which also derived out of the purple nested clade, showed a distribution predominantly restricted to the Black Sea coast.

In terms of climate, the average for annual precipitation in localities for clade P bats was lower than that for clade S bats (Table 5). The difference was statistically significant (*P *= 7.84*10^-33^, unpaired t-test, *N *= 33). With respect to the precipitation values, there was no overlap in the confidence intervals of the localities of clades S and P.

**Table 5:** Unpaired t-test results for climate differences (summer precipitation) between clades S and P.

Clade	Mean summer precipitation (mm)	t_crit _(df = 115)	s/√ n	Confidence Interval
P	396.3	1.98	16.7	(363.2, 429.4)
S	777.3	1.98	16.7	(744.2, 810.4)

### Microsatellites

In microsatellite analyses, none of the loci showed any evidence for linkage disequilibrium, after sequential Bonferroni corrections. The loci Mschreib4 and Mschreib5 were found not to be in Hardy-Weinberg equilibrium. A separate analysis of Hardy-Weinberg equilibrium, for each mtDNA clade, indicated that clade S was not in equilibrium and this probably was the cause for the overall disequilibrium when the two clades were analyzed together. The disequilibrium in these two loci was due to the presence of null alleles, and the microsatellite allele and genotype frequencies were adjusted, using the program Micro-Checker, prior to further data analyses.

A pattern of allelic differences was seen in loci Mschreib2 and Mschreib3, with Clade P individuals having relatively smaller sized alleles. There were private alleles in each population, and they had relatively low frequencies (less than 5%), except three alleles, which had frequencies over 10%. These were alleles 194 in locus Mschreib2 (31.3%), 133 in locus Mschreib3 (16.7%) in clade P, and 190 in locus Mschreib5 (13.4%) in clade S. There were also some differences in the frequencies of certain alleles, although they were not private alleles. For instance the allele size range series between 130–140 bp had a high frequency (33.4%) in clade P, however only two alleles in this series (133 and 137 bp) were found in clade S, in one individual each. For these two loci, based on the corrected number of private alleles, these differences were reflected as a higher number of private alleles for clade P, when compared to clade S (Additional File, Table AF7). Although to a smaller extent, the AMOVA of microsatellite loci, compared based on the two mtDNA clades also supported the break (Table 6), with a cumulative F_st _value 0.023 that was significantly greater than zero (*P *= 0.010, 1000 permutations). The R_st _value was comparable, but slightly higher (0.037), suggesting that F_st _slightly underestimated genetic differentiation when compared to R_st_.

**Table 6:** The results of global and locus-by locus AMOVAs, F_st _and R_st_

	**F**_st_			**R**_st_		
	
	F_st _Value	Within Pop. Variance	Among Pop. Variance	R_st _Value	Within Pop. Variance	Among Pop. Variance
**Global**	0.023	96	4	0.037	93	7
**Mschreib2**	0.013	99	1	0.082	92	8
**Mschreib3**	0.034	97	3	0.039	96	4
**Mschreib4**	0.009	98	2	0.000	100	0
**Mschreib5**	0.038	95	5	0.000	100	0
**D5**	0.020	98	2	0.089	91	9
**D6**	0.025	97	3	0.046	96	4

## Discussion

### Anatolian Suture Zone

Suture zones are often observed after postglacial range expansions where lineages that diverged in allopatry meet again [16]. In Europe, there are many different examples of an east/west division of lineages of animals and plants that diverged in separate refugia [52]. Genetic suture zones were also observed for bats in the Balkans and Anatolia [53-55]. In this study, for *M. schreibersii*, a similar suture zone, of two divergent mitochondrial clades, was seen within Anatolia. Although to a smaller extent, this differentiation in mtDNA was also reflected in the nuclear genome. The Bayesian estimate of the time to the most recent common ancestor of these two clades was 233,000 years BP (169,000–299,000 years BP), supporting a scenario of secondary contact after differentiation in isolation during the Pleistocene. The geographical distribution of clade S and clade P are in conformation with the previous study of Bilgin et al. [12], with clade S being found predominantly to the west, and clade P to the east. These results support the idea that the clade S differentiated in the Balkans, and clade P in refugia to the east of the Turkish border, in Armenia, Georgia or Iran during the glacial maxima in the last ice age. After the conditions became more hospitable, the two clades expanded into their current distribution, forming a suture zone in between, due to secondary contact.

Evaluation of whether the observed mtDNA differentiation was reflected in the nuclear genome, using microsatellites, did not provide any strong evidence for the presence of reproductive isolation and cryptic speciation. Although there was some statistically significant differentiation in microsatellites, the actual quantitative levels were low. Considering the nuclear locus that reflects the mitochondrial divide the best, Mschreib2, allele 194 was found only in individuals belonging to clade P with a frequency of 31.3%. Putting into consideration the point that none of these were homozygotes, 62.5% of the individuals had this allele. However, even with this frequency, it is not possible to assign the remaining 37.5% of individuals to either clade based on this locus. Another locus in *M. schreibersii*, Mschreib3, showed a similar break with all of the eighteen typed individuals except two in clade P having at least one allele smaller than 140 bases. However, still, two individuals in clade S had alleles in this size range, suggesting that a hypothesis of nuclear gene-flow among the two clades cannot be rejected. Hence it might be possible to conclude that although differentiation in refugia might have resulted in frequency differences in certain allele/locus combinations, this did not necessarily result in reproductive isolation.

Considering the details of the geographic distribution of each clade, clade S showed more of a coastal distribution, as opposed to clade P, which had a distribution restricted to inland. This kind of coastal vs. inland genetic differentiation has been observed in other species including mammals, e.g., Atlantic tree rats [56], lesser long-nosed bat [57], birds (e.g., swamp sparrow [58]), and plants (e.g., western white pine [59]). The significant differences between climate parameters with respect to distribution of these mitochondrial coastal and inland clades suggest some kind of climatic association for each clade. In Anatolia, coastal areas exhibit a more humid climate, in comparison to more steppe-like and drastically drier climates inland [4]. Precipitation can influence vegetation, insect density and composition in a region [60]. The distribution of the mitochondrial clades being closely associated with different rainfall intensities suggests that local adaptations to different habitats and climate regimes might have been a determinant of the differentiation of the species in the region. This kind of climatic association supports results from another congeneric species, *M. natalensis *in South Africa, which suggested an association between different biomes and population substructure [10].

In terms of other potential causal factors underlying this genetic break, none of the topographic barriers (the Marmara Sea, the Taurus Mountains or the eastern Anatolia Diagonal) seemed to be prominent impediments to dispersal. Individuals belonging to either clade S or clade P were found on either side of a putative barrier. Although AMOVA analysis showed some significant differences between regions as defined by geographic barriers (Φ_st _= 0.27), these differences were not as obvious as those seen when comparing clades S and P with each other (Φ_st _= 0.92). Also, the geographic differences did not hold up in all of the comparisons of the regions. For instance, significant differences were found between southern Anatolia and western Anatolia. However, no significant differences were found with comparisons of southern Anatolia and eastern Anatolia, even though Taurus Mountains delimits the border for southern Anatolia with both of the other regions. Hence although there is some genetic structure within the clades, the cause of this is probably not geographic barriers. The study of Bilgin et al. [12] had suggested that the Marmara and the Taurus were not likely to be impediments to dispersal, and the results of this study suggest that, in addition, the eastern Anatolian Diagonal does not seem to limit gene flow. Although some studies indicate that physical barriers can impede gene flow in bats [13,61,62], the results of this study also suggest that similar to two other bat species investigated in this region (*Rhinolophus euryale *[53], and *Myotis capacinnii *[54]), geographic barriers do not seem to limit gene flow for *M. schreibersii*.

### Scenarios of post-glacial expansion

Further analysis gave additional support to the idea of postglacial expansions as the best explanation for the mtDNA differentiation. Statistical parsimony networks for both clade S and clade P showed star-like phylogenies, which are theoretically indicative of population expansions [37]. For both of these clades, this pattern was supported by the *R*_2 _and *F*_*s *_statistics, the mismatch distributions of haplotypes and Bayesian skyline plots. Especially in clade S, certain haplotypes (S15, S3, S33 and S17) potentially represent nodes indicative of multiple bursts of population expansion.

The phylogenetic analyses showed no geographically associated structure within clade S or P. However, nested clade analysis [63] of the statistical parsimony networks showed subtle differentiation that delineated the details of possible historical dispersal routes. The nested cladograms for clade S showed four different second level nested clades (Figure 1b). The most ancestral haplotype, S15, showed a distribution almost exclusively restricted to the Balkans (Figure 1a). The central haplotype (S3) of the (light blue) nested clade 2.3, budding off from the ancestral nested clade 2.1 suggested a founder event from the Balkans to the Mediterranean coast and a subsequent range expansion of the (red) nested clade 2.4 along that coast. The (green) nested clade 2.2, which also derived out of the nested clade 2.1, showed a distribution predominantly restricted to the Black Sea coast, suggesting another migration route parallel to that coast. These results show the restriction of the most ancestral haplotype to the Balkans, followed by expansion and differentiation of different nested clades along the Black Sea and the Mediterranean coasts. This kind of post-glacial coastal expansion has been documented in relatively few taxa, with classical cases including human expansions such as colonization of eastern China [64] and western North America [65].

Using a Bayesian skyline plot approach, the timing of the onset of expansion of the entire clades S and P were found to be around 15,500 years ago and 4,500 years ago, respectively. These limits roughly correspond to the onset of deglaciation of continental ice sheets, after the last Heinrich event, around 15,000 years BP [66], and the stabilization of climate in the Holocene about 4,000 years BP to near today's temperature [7]. This suggests that the final range expansions of clades S and P occurred after the end of Pleistocene, in a time range when the climate started to get warmer. The initiation of the expansion of these clades corresponding to post-Pleistocene is supported by evidence from other species, which show similar post Pleistocene modes of population expansion [7].

This type of distribution is expected from a founder event matching a leptokurtic model of migration (with a greater number of long distance migrants) where the populations behind the initial migration front do not advance as readily [15,67]. NCA confirmed this with an explanation of contiguous and long-range expansion along the Black Sea and Mediterranean coasts respectively. This expansion pattern is also similar to that represented by the "grasshopper paradigm" where central and western Europe have been colonized from a Balkans' refuge, when the Iberian and Italian refugial populations were slowed down in the Pyrenees and the Alps [14]. However, in this case, rather than by another refugial population, the expansion of the most ancestral haplotypes seems to have been blocked by the founder population that had colonized the uninhabited regions.

Using the Bayesian skyline approach for the nested clades, the onset of expansion for nested clades 2.2 and 2.3 were calculated to have started approximately at 4,000 and 6,000 years ago, respectively. Assuming that S15 was in the Balkans when the other clades started to expand, these results imply that this single haplotype has been restricted to the Balkans for at least 6,000 years. This roughly corresponds to 1000 generations, assuming an average generation time of six years for *M. schreibersii *(DEH, Australia). The Balkans has been proposed to be a glacial refugium for many taxa [15], including bats [55]. Subsequent migrations of the other nested clades from the Balkans also seemed to follow geographic regions, such as the Black Sea and the Mediterranean with slightly different vegetation types and climate, and again suggest regional and climatic associations following migration events. This represents a form of extensive and long-term restriction of haplotypes and lineages to specific areas, and indicates that individuals belonging to the same matrilines have inhabited their respective geographic regions (Balkans, Black Sea coast, Mediterranean coast and inland) for hundreds of generations. Adaptations to local geographic conditions and climatic regimes can confer advantages to individuals, in terms of habituation to distribution and availability of roosts and resources [68], increased reproductive success, survival rate, reproductive output and recruitment [69]. The results of this study suggest that these adaptations and preferences might be conserved over hundreds of generations, affecting the intraspecific microevolution of a species and the distribution of its resulting diversity.

## Conclusion

Through combined analyses of the effects of various factors to the genetic differentiation of *M. schreibersii*, we were able to outline some of the details of the evolutionary history of this species in southeastern Europe and Anatolia. There was no evidence available that supported topographic barriers as determinants of genetic differentiation in this species. The results indicated mitochondrial genetic differentiation in glacial refugia, and subsequent range expansions coupled with various types of regional climate patterns. There was also evidence for long-term restriction of matrilines to these climatic regions, for hundreds of generations. Consequently, we were able to show how multiple biotic and abiotic events including glacial periods, climatic associations and historical dispersal patterns complemented each other in causing regional and local differentiation within a species. The next step along the lines of this research should include sampling in the winter, to see whether the observed genetic structure is maintained in the hibernation colonies or not.

## Authors' contributions

RB conceived the study, carried out the fieldwork and molecular genetic studies, performed statistical analyses and drafted the manuscript. AK coordinated the fieldwork and helped in drafting the manuscript. EÇ participated in the design of the study and performed statistical analyses. TD supervised the laboratory components of the study and helped in drafting the manuscript. JCM supervised the entire research. All authors read and approved the final manuscript.
